# An *emm*-type specific qPCR to track bacterial load during experimental human *Streptococcus pyogenes* pharyngitis

**DOI:** 10.1186/s12879-021-06173-w

**Published:** 2021-05-21

**Authors:** Loraine V. Fabri, Kristy I. Azzopardi, Joshua Osowicki, Hannah R. Frost, Pierre R. Smeesters, Andrew C. Steer

**Affiliations:** 1 Tropical Diseases Research Group, Murdoch Childrens Research Institute, Melbourne, Victoria Australia; 2 Department of Paediatrics, Universit Libre de Bruxelles, Brussels, Belgium; 3 Department of Paediatrics, University of Melbourne, Melbourne, Victoria Australia; 4 Infectious Diseases Unit, Department of General Medicine, The Royal Childrens Hospital Melbourne, Melbourne, Victoria Australia; 5 Academic Children Hospital Queen Fabiola, Universit Libre de Bruxelles, Brussels, Belgium; 6 Molecular Bacteriology Laboratory, Universit Libre de Bruxelles, Brussels, Belgium

**Keywords:** Pharyngitis, Human challenge study, qPCR, Nucleic acid extraction, *Streptococcus pyogenes*

## Abstract

**Background:**

*Streptococcus pyogenes* causes a profound global burden of morbidity and mortality across its diverse clinical spectrum. To support a new controlled human infection (challenge) model seeking to accelerate *S. pyogenes* vaccine development, we aimed to develop an accurate and reliable molecular method for quantifying bacterial load from pharyngeal swabs collected during experimental human pharyngitis.

**Methods:**

Combined sequential RNA+DNA extraction from throat swabs was compared to traditional separate RNA-only and DNA-only extractions. An *emm*-type specific qPCR was developed to detect the *emm*75 challenge strain. Results from the qPCR were compared to culture, using throat swab samples collected in a human challenge study.

**Results:**

The qPCR was 100% specific for the *emm*75 challenge strain when tested against a panel of *S. pyogenes emm*-types and other respiratory pathogens. Combined RNA+ DNA extraction had similar yield to traditional separate extractions. The combined extraction method and *emm*75 qPCR had 98.8% sensitivity compared to culture for throat swabs collected from challenge study participants.

**Conclusions:**

We have developed a reliable molecular method for measuring *S. pyogenes* bacterial load from throat swabs collected in a controlled human infection model of *S. pyogenes* pharyngitis.

**Trial registration:**

NCT03361163 on 4th December 2017.

**Supplementary Information:**

The online version contains supplementary material available at 10.1186/s12879-021-06173-w.

## Background

*Streptococcus pyogenes* is a human-only pathogen with a diverse clinical spectrum including severe syndromes responsible for more than 500,000 deaths worldwide each year [1, 2]. Hope for a vaccine to prevent *S. pyogenes* infections and their complications predates the identification of the bacterium in the late nineteenth century, when the disease spectrum was loosely considered under the historical umbrella term of scarlet fever. While vaccination is viewed as the intervention most likely to achieve sustainable diseases control, development has been impeded by regulatory, commercial, and scientific obstacles, including critical knowledge gaps regarding host-pathogen interactions [3].

Controlled human infection (human challenge) studies recruit carefully screened volunteers who are deliberately infected under highly controlled conditions with a well-characterised strain of an infectious agent. Human models contribute to vaccine development as platforms for direct evaluation of vaccine protective efficacy and for detailed studies of bacterial pathogenesis and human immunity, spotlighting potential correlates of protection [4]. A multinational collaborative group established the Controlled Human Infection for Vaccination Against *Streptococcus pyogenes* (CHIVAS) model of experimental human *S. pyogenes* pharyngitis in healthy adult volunteers in Melbourne, Australia. The first CHIVAS study (CHIVAS-M75, ClinicalTrials.gov number NCT03361163) sought to determine a dose of a carefully selected *S. pyogenes* M75 strain (GenBank CP033621) required to cause pharyngitis in at least 60% of participants following direct inoculation of the pharynx by a swab [5,7]. The M75 strain was isolated from a child with acute pharyngitis in Melbourne. It was selected for use as a challenge strain according to a rationale prioritising clinical relevance and participant safety, and was extensively characterised by whole genome sequencing, in vitro assays, and in an animal model of invasive infection [5, 8].

The CHIVAS model includes serial collection of throat swabs for detection and quantification of pharyngeal *S. pyogenes* colonisation following the challenge procedure. We aimed to develop a robust molecular method for quantifying colonisation by the challenge strain from serial throat swabs. To study the dynamics of infection and clearance, we developed a reliable, rapid, scalable, and highly sensitive and specific method to overcome some of the limitations of traditional culture-based methods [9]. Here, we describe the performance of a type-specific *emm*75 real-time quantitative PCR (qPCR) in testing DNA extracted from throat swabs collected during experimental human *S. pyogenes* pharyngitis in the CHIVAS-M75 trial.

## Methods

### Collection of throat swabs

Two throat swabs (FLOQswabs^(R)^, CopanItalia SpA) were collected on the day before challenge, approximately every 12h during an inpatient period of up to 6days, and at outpatient follow-up visits (1week, 1month and 3months after discharge). One swab was stored in 1ml eSwab (Copan Italia SpA)solution for culture onto horse blood agar (HBA) and the other swab was placed into 2ml eNat (Copan Italia SpA)nucleic acid preservation medium (guanidine thiocyanate-based solution) and stored at 20C for up to 6 months.

### Preparation of genomic DNA from different bacterial species

To assess the specificity of the *emm*75 qPCR assay to distinguish the *S. pyogenes* M75 challenge strain from other common upper respiratory tract pathogens, pellets of *S. pyogenes*, *Streptococcus pneumoniae*, *Staphylococcus aureus* and *Haemophilus influenzae* were harvested by centrifugation at 2,800 rcf from 25ml mid-log phase cultures, and colony-forming units (CFU) were enumerated by plating. Bacterial cultures were centrifuged and genomic DNA (gDNA) was isolated from pellets using the DNeasy Blood and Tissue kit (Qiagen) according to the manufacturers modifications for Gram-positive bacteria. Briefly, each bacterial pellet was incubated for 45m at 37C with 180l of enzymatic lysis buffer (lysozyme 20mg/ml, 20mM Tris-HCl pH8.0, 2mM EDTA and 1.2% v/v Triton-X), then DNA from the lysate was purified on column as per manufacturer instructions. All DNA was eluted from purification columns in a final volume of 50l. Concentration of eluted DNA was adjusted to 10^4^ Genome Equivalents (GE)/l for all *S. pyogenes* strains (assuming one genome per CFU and *S. pyogenes* genome size of 1.8Mb). DNA from *S. pneumoniae* (clinical isolate, serotype 5), *S. aureus* (ATCC 25923) and *H. influenzae* (ATCC 10211) was adjusted to 10^5^ GE/l according to their respective genome sizes (2Mb, 2.8Mb and 1.8Mb) [10,12]. Control human pharyngeal DNA was extracted from a healthy adult donor, using the DNeasy Blood and Tissue kit according to the instructions for mammalian cultured cells. For qPCR sensitivity testing, gDNA was prepared from a dilution series of M75 broth culture from 10^0^ to 10^7^CFU/ml.

### Separate and combined sequential extraction of RNA and DNA

We compared RNA-only and DNA-only extractions using separate spin columns with a protocol for combined sequential RNA and then DNA extraction from the same spin column, as described by Kerllen Martins [13]. In the combined RNA+DNA method, RNA was initially isolated using a a single RNeasy Mini kit column with RNase-free water (pH4.5), then DNA waseluted off the same column with elution buffer from the DNeasy Blood and Tissue kit, pH7.5 (Additionalfile1: Method S1). RNA derived using either protocol was treated with the TURBO DNA-free kit (Invitrogen) to enzymatically digest contaminating DNA. The RNA was then assessed for purity and concentration by spectrophotometry using the NanoDrop 2000 system (ThermoFisher) and the 4200 TapeStation (Agilent) prokaryotic RNA High sensitivity ScreenTape Assay.

### Evaluation of extraction methods

To examine the effect of chemical pre-lysis prior to extraction, 200l volumes of eNat medium containing *S. pyogenes* M75 broth culture at three concentrations (910^2^/10^4^/10^6^CFU/ml) was stored at 20C for 7days. Using the combined RNA+DNA method, extraction was completed with and without a pre-lysis step. An additional extraction was done without pre-lysis using a larger 600l volume of spiked eNat at a single concentration (910^4^ CFU/ml). To compare separate and combined extractions, a dilution series of *S. pyogenes* M75 broth culture (determined by spread-plate dilution) was combined with eNat medium for final concentrations of 310^1^/10^2^/10^4/^10^6^CFU per ml. These eNat tubes were frozen at 20C for 6weeks and extraction was performed using the RNA+DNA method without pre-lysis.

### Quantitative PCR

To assess the quality of simultaneously extracted RNA and DNA, the RNA was reverse transcribed using the Protoscript II Reverse Transcriptase kit (NEB) and the resulting cDNA and extracted gDNA were tested by qPCR. Relevant primer and probe sequences are listed in Table1. The *emm*75 assay (IDT) targets a 123bp stretch of the *emm*75 hypervariable region (from 146 to 268bp inclusive). Reactions using FAM-fluorescent probes (*emm*75 and *speB*) were performed using the GoTaq Probe qPCR Master Mix (Promega). GoTaq SYBR green qPCR Master Mix (Promega) was used for *gyrA, lytA, gltB, hpd3* and mammalian mitochondrial rRNA. Reactions were prepared using 4l of template DNA or cDNA in a final volume of 20l with primers and probes at a final concentration of 250nM. Duplicate runs were completed on an AriaMX qPCR instrument (Agilent) with the following fast 2-step conditions: initial hot start of 1cycle at 95C, followed by 40cycles of denaturation at 95C for 3s and combined annealing/extension at 60C for 30s. No template control (NTC) and no reverse transcriptase (NRT) controls were included in each assay.

**Table 1:** Primers and probes used in this study

Target	Gene	Primer and probe sequences (5-3)	Reference
*Streptococcus pyogenes* M75	*emm*75	Probe: 56-FAM/TGGAAAAGT/ZEN/GAAAATGATGAGCTTCGGG/3IABkFQ	This study
		F:AGTTACCATATGAAGCACGATACAA	
		R:GTTCTTCTAATCTCGTAGTCTTACCT	
	*speB*	Probe: FAM-CGGCGCAGGCGGCTTCAAC-BHQ1	[8]
		F: CTAAACCCTTCAGCTCTTGGTACTG	
		R: TTGATGCCTACAACAGCACTTTG	
	*gyrA*	F: CGACTTGTCTGAACGCCAAA	[14]
		R: GTCAGCAATCAAGGCCAACA	
*Streptococcus pneumoniae*	*lytA*	F: ACGCAATCTAGCAGATGAAGCA	[15]
		R: TCGTGCGTTTTAATTCCAGCT	
*Staphylococcus aureus*	*gltB*	F: CGGGTTAGGTGAATTGATTGTTTTAT	[16]
		R: CGCATTTGAGCTGAAGTTG	
*Haemophilus influenzae*	*hpd3*	F: GGTTAAATATGCCGATGGTGTTG	[17]
		R: TGCATCTTTACGCACGGTGTA	
Mammalian mitochondrial rRNA	16S *mt rRNA*	F: CGACCTCGATGTTGGATCAG	[18]
		R: GAACTCAGATCACGTAGGACTTT	

### qPCR sensitivity and limit of detection

To assess the limit of detection (LOD) for the *emm*75 qPCR we prepared template using a 10-fold dilution series of *S. pyogenes* M75 gDNA (10^0^10^7^CFU/ml). Ct variation at the lowest template concentration (10^0^) was high so these values were excluded from calculation of the standard curve. The lowest dilution to produce consistent Ct values was 10^1^CFU/ml, equivalent to 40 GE of *S. pyogenes* M75, considered as the LOD of the *emm*75 qPCR. The *emm*75 assay was compared to a previously published qPCR targeting *speB*, a virulence factor in the core chromosome of *S. pyogenes* and highly conserved across different *S. pyogenes emm*-types [8].

### qPCR specificity

To assess the *emm*-type and species specificity of the *emm*75 qPCR assay, 14 other *S. pyogenes* strains were also tested, representing each of the 11 major *emm* clusters (E16, D2, D4, AC35), and two single protein clades [19]. Other bacteria commonly detected in the human oropharynx were also tested. The *emm*75 primer and probes were blasted against the online NCBI genome database (https://blast.ncbi.nlm.nih.gov/Blast.cgi) to confirm the absence of homologous sequences, besides the *emm*75 allele.

### CHIVAS-M75 study samples

The CHIVAS-M75 study protocol has been described in detail elsewhere [6]. The study was approved by the Alfred Hospital Human Research Ethics Committee (500/17). Twenty healthy adult volunteers were screened for *S. pyogenes* using culture and a rapid molecular point-of-care test (Abbott ID NOW Strep A 2, formerly Alere i) in the 28days prior and then again in the 12h before direct swab application of *S. pyogenes* M75 (at a dose of 10^5^ CFU/ml)to the pharynx [20].

### Throat swab cultures using eSwab

Each eSwab collected in the CHIVAS-M75 trial (*n*=192) was plated onto HBA at an external contract clinical laboratory according to the manufacturer instructions (Copan) within 48h of collection and incubated at 37C in 5% CO_2_. The next day, growth of -hemolytic colonies was visually estimated according to guidelines developed in our laboratory: 10-fold dilutions of *S. pyogenes* M75 in broth culture (range 010^5^CFU/ml) were prepared and an eSwab dipped into each dilution for 30s then plated on HBA for single colonies following the eSwab protocol. Semi-quantitative growth scores were established based on the results of this test: 0=no growth; 1=light; 2=moderate; 3=heavy; 4=profuse (Additionalfile2: Figure S1). At the external laboratory, -hemolytic colonies were selected and confirmed as *S. pyogenes* by MALDI-TOF mass spectrometry (Bruker Microflex) [21]. Colonies were re-plated for purity then stored at 80C. Pure vials were sent to our laboratory and all were confirmed as *emm*75 by gene amplification and Sanger sequencing [22].

### Throat swabs for molecular analyses using eNat

Combined RNA+DNA extraction was done from 192 eNat swabs, with laboratory staff blinded to the clinical trajectory of the source participants in the CHIVAS-M75 trial. Genomic DNA was quantified by qPCR and compared to the eSwab semi-quantitative growth scores. Use of the extracted RNA to study gene bacterial and host expression (e.g., by reverse transcription-qPCR) will be the object of future work and is not explored in the methodology described herein. For determination of bacterial load in the eNat samples by *emm*75 qPCR: A set of standards were generated using 10-fold dilutions of *S. pyogenes* M75 (ranging from 10^1^ to 10^7^CFU/ml) which was then used to spike eNat vials containing oropharyngeal swabs from two healthy adult donors. Standards were frozen for 7days, thawed to mimic handling of samples from the clinical trial, and DNA extracted using the combined RNA+DNA protocol. These standards were included on each 96-well plate to generate a seven-point standard curve, along with a negative no template control (NTC). Each reaction was performed in duplicate. Cycle thresholds (Ct) were ascertained with Agilent AriaMX 1.7 software using R fluorescence and automated thresholds. Reliable *emm*75 detection occurred at Cts 35, considered thereafter as the limit of detection (Additionalfile3: Figure S2)*.*

### Data analysis

Analyses were performed using GraphPad Prism 8 software. Efficeincy of the qPCR was calculated with Agilent AriaMX software from a formula assigning 100% when 10-fold serial dilutions of template cause an increase of 3.3cycles (i.e. linear regression slope=3.3) [23]. M75 bacterial load was expressed as log_10_ *emm*75 genome equivalents (GE) per ml of eNat medium. Separate or combined column extractions were compared by paired t-tests and the non-parametric Spearman coefficient was used to consider correlation between plate growth scores and *emm*75 GE [8].

## Results

### qPCR sensitivity

**Fig. 1:**
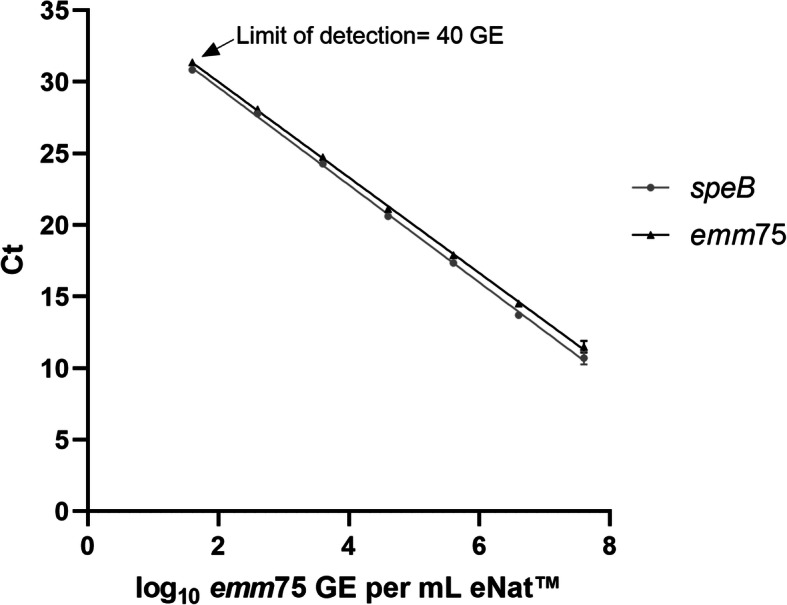
Performance of the *emm*75 qPCR. qPCR targeting the *emm75* or *speB* gene was performed on genomic DNA isolated from 10-fold dilutions of *S. pyogenes* M75. The *speB* qPCR was used to provide a benchmark for the performance of the *emm75* qPCR, using FAM-conjugated probes for both reactions. Each point represents the mean and standard deviation (SD) of duplicate reactions

The *emm*75 qPCR assay had superior efficiency of 99.2% based on a slope of 3.3 (R^2^=0.999) (Fig.1). In comparison, the *speB* assay had 96.5% efficiency (slope=3.4, R^2^=0.999). Ct values for the *speB* assay were marginally lower than the *emm*75 assay (mean difference 0.56 Ct, 95% CI [0.40, 0.73]), however the assays had similar levels of sensitivity, with lower limits of detection of 40 GE/ml when corrected for efficiency.

### qPCR specificity

The *emm*75 qPCR was 100% specific within the detectable concentration range, producing an amplification signal for assays containing *emm*75 template and no signal for assays containing non-*emm*75 *S. pyogenes*, *S. pneumoniae*, *S. aureus*, *H. influenzae* or DNA extracted from pharyngeal swabs from a healthy adult. The *speB* qPCR was 100% specific, being positive for all 15*S. pyogenes* templates and negative for the other bacterial species tested (all 4 species were positive in relevant species-specific assays) (Additionalfile4: Table S1).

### Evaluation of extraction methods

**Fig. 2:**
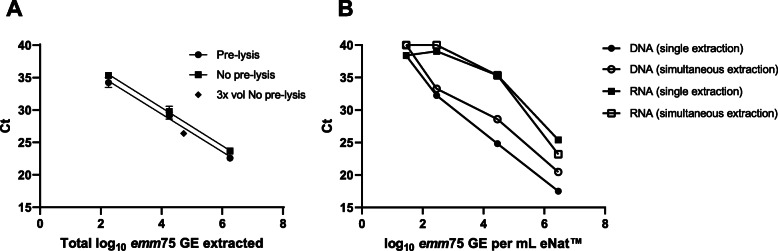
Optimisation of the method for isolation of DNA and RNA from throat swabs in eNat. **a** Pre-lysis versus No pre-lysis extractions using the combined RNA+DNA approach. eNats were spiked with *S. pyogenes* M75 at three concentrations and 200l aliquots (1.810^2^, 1.810^4^, 1.810^6^ total GE) underwent DNA extraction using a chemical Pre-lysis step () or No pre-lysis (). A larger 600l aliquot (3x vol) of eNat medium spiked with M75 (5.410^4^ total GE) was processed without pre-lysis (). Data shown in total GE to account for differences in input bacteria. Each point represents the mean and SD of two eNats tested in duplicate qPCRs. **b** eNats containing a range of M75 were used to test separate column extractions (RNA-only or DNA-only ) versus combined RNA+DNA isolation (RNA**** and DNA****). The *emm75* qPCR was used to assess DNA whilst RNA was converted to cDNA and tested in a SYBR Green qPCR for the housekeeping gene, *gyrA.* Each point represents the mean and SD of three eNats tested in duplicate qPCRs targeting the *emm75* gene

Effective extraction of DNA from Gram-positive bacteria such as *S. pyogenes* usually requires an enzymatic peptidoglycan digestion step to lyse the cell wall. The eNat medium contains lysing agents, so that a pre-lysis incubation step or addition of supplemental lytic agents may not be required. We performed DNA extraction with and without an enzymatic pre-lysis step to assess whether this influenced DNA yield in the *emm*75 qPCR. At each dilution tested, pre-lysis generated lower Cts than extraction without pre-lysis (mean difference 0.9458 Ct, *P* value=0.03, 95% CI [0.28, 1.61]; Fig.2a). When a higher volume of eNat medium was processed through the column without pre-lysis (600l versus 200l, both 910^4^ CFU/ml) the mean Ct was 26.39 (Fig. 2a,)- lower than the extrapolated 27.27 for the equivalent bacterial load with pre-lysis (pre-lysis linear regression: Ct=[2.914.73]+41.05). Although not tested across a range of input bacteria CFUs, this finding suggests that increasing the processing volume of eNat is more effective for increasing DNA yield than applying a pre-lysis step.

DNA yield from the combined RNA+DNA extraction protocol was lower than the DNA-only method as determined by UV absorbance and qPCR (mean difference 2.4 Ct, *P* value=0.03, 95% CI [0.38, 4.3]; Fig. 2b, Additionalfile5: Table S3). As expected, for RNA (and resulting cDNA) both extraction protocols achieved similar Ct values and limits of detection in a qPCR assay targeting the *S. pyogenes* housekeeping gene *gyrA* (mean difference 0.08 Ct, P value 0.93, 95% CI [2.57 to 2.73]; Fig. 2b). NanoDrop and TapeStation systems did not produce consistent estimations of RNA concentration and quality due to low concentration (Additionalfile6: Table S3). As results were similar and the combined extraction protocol reduced both time and cost of extraction, this method was used for the CHIVAS-M75 study samples.

### Detection of *emm*75 bacterial load in vivo by qPCR

**Fig. 3:**
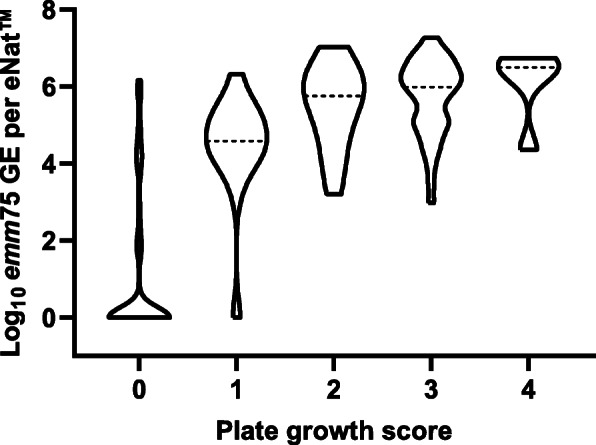
Correlation between culture and qPCR results. The number of *emm*75 genome equivalents (GE), extrapolated from qPCR Ct values, was compared to plate growth scores derived by culture. The median log10 *emm*75 GE of each score is represented by the dotted line (---), whilst the width of the shape reflects the density of data at the respective values

During the CHIVAS-M75 study, 20 participants were challenged with 10^5^ CFU/ml of M75,and 192 paired throat swabs (between 8 and 14 time points per participant) were collected for culture (eSwab) and molecular testing (eNat). There was high correlation between culture and qPCR results (Spearman correlation R=0.8058, *P*<0.001; Fig.3).

Culture and qPCR results were concordant (positive or negative) for 171 of 192 evaluable sample pairs (89.1%) (Table2). All paired swabs taken prior to challenge were negative by both qPCR and culture. One culture-positive eSwab was negative by qPCR, giving a sensitivity of 98.8% (95% CI [97.3, 100]) for the qPCR assay. Swabs for 20 time points were qPCR positive and culture negative, including 16 collected after antibiotic treatment and 4 from the first 48h after challenge in participants whose subsequent swabs were culture positive (Additionalfile7: Table S4). All culture-positive swabs were verified as *S. pyogenes* M75 by qPCR and *emm* typing.

**Table 2:** Test results for culture and qPCR from throat swabs

		**Culture**		**Total PCR**
		**+**	****	
**PCR**	**+**	83	20	103 (53.6%)
	****	1	88	89 (46.4%)
**Total culture**		84 (43.7%)	108 (56.3%)	192 (100%)

A positive qPCR result is defined by a Ct35 as determined by a seven-point standard curve run on each qPCR. A positive culture result is defined by a plate growth score1

## Discussion

We have developed a sensitive and specific quantitative PCR assay for measurement of *S. pyogenes* M75 from throat swabs in a human infection study of *S. pyogenes* pharyngitis. We have shown that the qPCR is specific for the *emm*75 gene encoded by the challenge strain with a high level of analytical performance for efficiency, specificity, and sensitivity. A lysis step prior to extraction of DNA from throat swabs stored in eNat was not critical for DNA extraction, attributable to bacterial lysis by the proprietary reagents in the eNat medium. There were only minor differences in yield between separate RNA-only and DNA-only extractions and combined RNA+DNA isolation using a single RNeasy column. The combined approach was simpler, faster, and enabled processing of the entire 2ml eNat volume using a single column, reducing cost. Although not the primary aim of this study, the ability to extract quality cDNA from RNA, in addition to DNA for bacterial load quantification, increases the potential value of this technique. Finally, we compared qPCR results to semi-quantitative culture scores ('no growth' score of 0 is quantitative), showing the performance of the assay for its intended use- to measure bacterial load from throat swabs collected in a human challenge study.

Several studies have shown that qPCR is highly sensitive compared to culture and can be used to accurately quantify bacterial load during natural *S. pyogenes* pharyngitis [8, 24,27].

The *emm*75 qPCR assay we developed is rapid, scalable, and type-specific. The entire process from DNA extraction to qPCR can provide a fully quantitative result in 4h and can be performed on swabs stored in eNat solution for at least 6months without loss of DNA yield. The contract clinical trials facility for the CHIVAS-M75 study was offsite, with limited local laboratory capabilities, so that stability during storage and transport was critical. In comparison, the combined processes for *S. pyogenes* M75 enumeration and *emm*-typing by traditional methods requires at least 7days (initial culture, purity plating, speciation, group typing, extraction of DNA, *emm* gene sequencing and analysis).

Historically, human infection studies for bacterial pathogens (including *S. pyogenes* pharyngitis studies in the 1970s) have used traditional bacterial culture methods to measure bacterial load during experimental infection [28,31]. Several non-human primate models of *S. pyogenes* pharyngitis have performed PCR for sample analysis (mainly for gene expression or to confirm the *emm*-type), however plate enumeration was the principal method to quantify bacterial load [32,35].

We found a strong correlation between *emm*75 qPCR Ct values and plate growth scores. Discrepancies were noted in 20 swab pairs that were qPCR positive and culture negative, early after challenge as bacterial load was increasing or after initiation of antibiotic treatment when *S. pyogenes* M75 colonisation was waning. These data likely reflect the lower limit of detection and higher sensitivity of qPCR compared to culture, and detection of non-culturable bacterial elements [8, 36]. Reduced sensitivity of bacterial culture following antibiotic treatment is common [37]. Molecular methods have also shown higher sensitivity in studies using other human pathogens, especially following antibiotic treatment [38, 39]. There was a single pair of discordant qPCR negative and culture positive swabs, collected after antibiotic treatment. Subsequent swabs were negative by both techniques. This discrepancy is likely explained by variation in swab collection. All volunteers returned a negative qPCR result within 7days of discharge, showing the usefulness of qPCR in differentiating persistent colonisation from relapse or re-infection during the period of outpatient follow up.

This study has several limitations. The categorical and semi-quantitative plate growth scoring system clouds direct comparison to continuous qPCR data. The eSwabs for culture were collected at the off-site commercial trials facility and transferred to a contracted laboratory for culture according to diagnostic protocols and workflows with semi-quantitative enumeration as an additional step. The eNat swabs were immediately frozen and later transported to our research laboratory for batched molecular testing. This variation could conceivably have contributed to differences between culture and qPCR results. Detection of bacterial DNA from non-viable bacteria may cause false-positive results by qPCR. For the CHIVAS-M75 trial, qPCR detection was at least equivalent to culture. Even more precise bacterial quantification could be overcome if desired in future studies using a marker of viability. As discussed, further work remains to be done with RNA extracted from throat swabs collected in the CHIVAS-M75 trial.

## Conclusions

We have developed a reliable, rapid, scalable, and highly sensitive and specific molecular method to quantify pharyngeal colonisation by *S. pyogenes* M75 in a controlled human infection model.

## Supplementary Information

**Additional file 1: Method S1.** The protocol for combined RNA and DNA extraction from eNat medium containing participant throat swabs.

**Additional file 2: Figure S1.** Visual guidelines for semi-quantitative scoring of the *S. pyogenes* M75 challenge strain from eSwabs.

**Additional file 3: Figure S2.** Standard curve for determination of *emm*75 GE in eNats by qPCR. Seven-point standard curves were included on each *emm*75 qPCR plate and used to extrapolate bacterial load for eNat throat swabs from the CHIVAS-M75 trial, expressed as *emm*75 genome equivalents (GE) per ml of eNat medium. The dotted line represents the limit of detection Ct35. Mean and standard deviation shown from 7 duplicate runs.

**Additional file 4: Table S1.** Specificity of the *emm*75 qPCR. *S. pyogenes* from different *emm*-clusters and other respiratory pathogens were used to demonstrate the specificity of the *emm*75 qPCR. Each value is the mean of duplicate reactions. Samples with no provided Ct value failed to cross the fluorescence threshold by the 40th (last) cycle of the assay and all samples with Ct values greater than 35 were considered negative. No template control was included and this did not cross the fluorescence threshold by the 40th cycle.

**Additional file 5: Table S2.** *Emm*75 qPCR and NanoDrop DNA readings. Comparison between DNA-only and single column combined extraction methods (RNA eluted first). Refer to Fig. 2b DNA readings.

**Additional file 6: Table S3.** *gyrA* RT-qPCR and NanoDrop RNA readings. Comparison between RNA-only and single column combined extraction methods (RNA eluted first). Refer to Fig. 2b RNA readings.

**Additional file 7: Table S4.** Detailed timeline of discrepancies between culture and qPCR results. Twenty swabs were positive by *emm*75 qPCR and negative by culture. These swabs were either collected within 48h after inoculation (*N*=4) or within 36h after initiation of antibiotic treatment (*N*=16). 0, pre challenge; +24, 24h post challenge; Abx, antibiotics; +1 w, 1week post discharge; +1m, 1month post discharge; +3m, 3months post discharge.

## Data Availability

All data generated and analysed during the study are included in this published article and its additional supplementary files. Raw datasets are available from the corresponding author on reasonable request.

## Abbreviations

cDNA: Complementary DNA
CHIVAS: Controlled Human Infection for Vaccination Against *Streptococcus pyogenes*
CI: Confidence interval
Ct: Cycle thresholds
gDNA: Genomic DNA
GE: Genome equivalents
NRT: No reverse transcriptase
NTC: No template control
qPCR: Real-time quantitative polymerase chain reaction
*S. pyogenes*: *Streptococcus pyogenes*
*S. pyogenes* M75: *Streptococcus pyogenes* M75 challenge strain (ID 611024)
