# Supplementary material for: An emm-type specific qPCR to track bacterial load during experimental human Streptococcus pyogenes pharyngitis
Source: BMC Infect Dis. 2021 May 21;21:463. doi: 10.1186/s12879-021-06173-w (PMC8138111; doi:10.1186/s12879-021-06173-w)
Supplement: Supplementary file 1 — Additional file 1: Method S1. The protocol for combined RNA and DNA extraction from eNat medium containing participant throat swabs. [file 12879_2021_6173_MOESM1_ESM.pdf]

Method S1. The protocol for combined RNA and DNA extraction from eNat™ medium containing participant throat swabs.

1. Remove eNat™ vials from -20°C storage and allow to thaw at room temperature.
2. Vortex thawed eNat™ samples for 5 s, remove and discard the swab.
3. Add 70% (v/v) volume of 100% ethanol (molecular analysis grade) to each sample and mix by inversion 3 times (i.e, for 2 ml eNat add, 1.4 ml of 100% ethanol).
4. Transfer 710 µL to RNeasy® spin column placed in a collection tube. Centrifuge at 13,000 rcf for 30 s. Discard flow-through and replace the spin column in the same collection tube. Repeat this step for a total of 5 times, so that the entire volume of sample is transferred onto the spin column. Place the column in a new collection tube at the end of the last step.
5. Add 700 µL of Buffer RW1 to the spin column. Centrifuge at 13,000 rcf for 30 s. Discard flow-through and replace the spin column in the same collection tube.
6. Add 500 µL of Buffer RPE to the spin column. Centrifuge at 13,000 rcf for 30 s. Discard flow-through and replace the spin column in the same collection tube.
7. Add 500 µL of Buffer RPE to the spin column. Centrifuge at 13,000 rcf for 2 m. Discard flow-through and place the spin column in a new collection tube.
8. Centrifuge at 13,000 rcf for 1 m. Discard flow-through and place the spin column in a 1.5 mL DNase and RNAse-free tube, clearly labelled "RNA".
9. Add 50 µL RNAse-free water (pH 4.5) directly onto the spin column membrane. Stand for 5 m at room temperature and centrifuge at 13,000 rcf for 1 m. Place the column in a new 1.5 mL DNAse and RNAse-free tube labelled "DNA". Store RNA at -80°C.
10. Add 50 µL of prewarmed (55°C) DNA elution buffer (pH 7.5) directly onto the spin column membrane and incubate for 5 m at 55°C. Centrifuge at 4 000 rcf for 3 m. Store DNA at -80°C.
