# Supplementary material for: An emm-type specific qPCR to track bacterial load during experimental human Streptococcus pyogenes pharyngitis
Source: BMC Infect Dis. 2021 May 21;21:463. doi: 10.1186/s12879-021-06173-w (PMC8138111; doi:10.1186/s12879-021-06173-w)
Supplement: Supplementary file 2 — Additional file 2: Figure S1. Visual guidelines for semi-quantitative scoring of the S. pyogenes M75 challenge strain from eSwabs. [file 12879_2021_6173_MOESM2_ESM.pdf]

Figure S1. Visual guidelines for semi-quantitative scoring of the *S. pyogenes* M75 challenge strain from eSwabs™

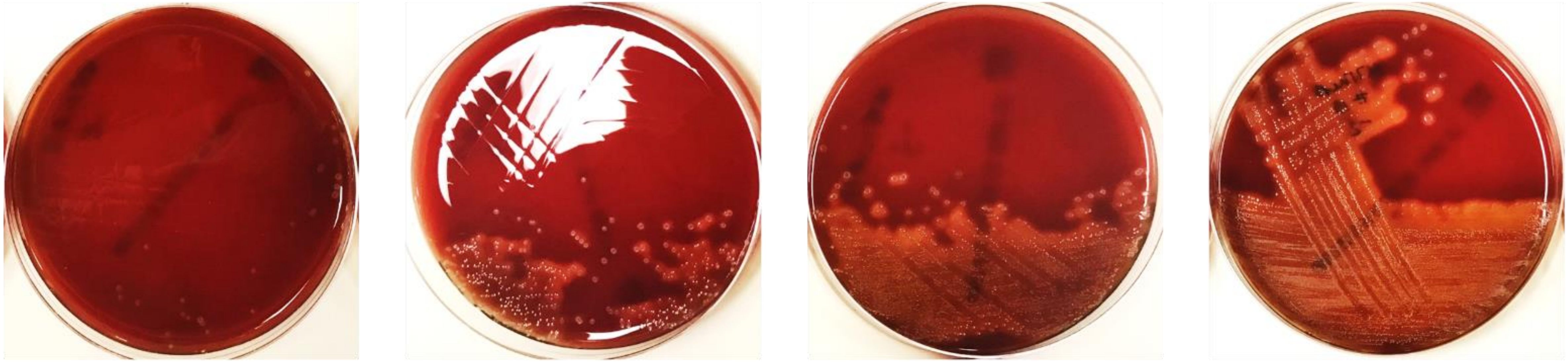

Light
Score 1

Moderate
Score 2

Heavy
Score 3

Profuse
Score 4
