# Supplementary figures and images for: An emm-type specific qPCR to track bacterial load during experimental human Streptococcus pyogenes pharyngitis
Source: BMC Infect Dis. 2021 May 21;21:463. doi: 10.1186/s12879-021-06173-w (PMC8138111; doi:10.1186/s12879-021-06173-w)

# Standard curve for determination of *emm75* GE in eNats™ by qPCR

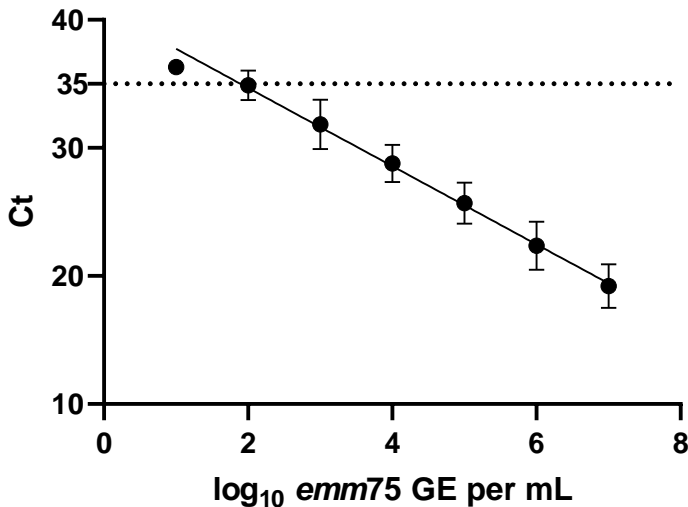

Supplement: Supplementary file 3 — Additional file 3: Figure S2. Standard curve for determination of emm75 GE in eNats by qPCR. Seven-point standard curves were included on each emm75 qPCR plate and used to extrapolate bacterial load for eNat throat swabs from the CHIVAS-M75 trial, expressed as emm75 genome equivalents (GE) per ml of eNat medium. The dotted line represents the limit of detection Ct35. Mean and standard deviation shown from 7 duplicate runs. [file 12879_2021_6173_MOESM3_ESM.pdf]
