# Supplementary material for: An emm-type specific qPCR to track bacterial load during experimental human Streptococcus pyogenes pharyngitis
Source: BMC Infect Dis. 2021 May 21;21:463. doi: 10.1186/s12879-021-06173-w (PMC8138111; doi:10.1186/s12879-021-06173-w)
Supplement: Supplementary file 4 — Additional file 4: Table S1. Specificity of the emm75 qPCR. S. pyogenes from different emm-clusters and other respiratory pathogens were used to demonstrate the specificity of the emm75 qPCR. Each value is the mean of duplicate reactions. Samples with no provided Ct value failed to cross the fluorescence threshold by the 40th (last) cycle of the assay and all samples with Ct values greater than 35 were considered negative. No template control was included and this did not cross the fluorescence threshold by the 40th cycle. [file 12879_2021_6173_MOESM4_ESM.pdf]

	emm-cluster	Cycle threshold (Ct)					
		***emm*75**	***speB***	***lytA***	***gltB***	***hpd3***	**mt rRNA**
S. pyogenes M75 challenge strain (ID 611024) ***emm*75**	E6	19.45	18.06				
***emm* 67**		negative	20.21				
***emm* 81**		negative	19.68				
***emm* 4.5**	E1	negative	20.08				
***emm* 104**	E2	negative	18.94				
***emm* 9**	E3	negative	19.18				
***emm* 22**	E4	negative	19.78				
***emm* 170**	E5	negative	20.20				
***emm* 71**	D2	negative	19.02				
***emm* 41.2**	D4	negative	19.61				
***emm* 1**	AC-3	negative	22.48				
***emm* 12**	AC-4	negative	19.44				
***emm* 3**	AC-5	negative	19.04				
***emm* 6**	Single protein Clade Y	negative	19.34				
***emm* 55**	Single protein outlier	negative	19.95				
S. pneumoniae (clinical isolate, serotype 5)		negative		10.35			
S. aureus (ATCC 25923)		negative			18.36		
***H. influenzae* type b** (ATCC 10211)		negative				17.15	
**Healthy human throat swab**		negative					13.53
