# Supplementary material for: An emm-type specific qPCR to track bacterial load during experimental human Streptococcus pyogenes pharyngitis
Source: BMC Infect Dis. 2021 May 21;21:463. doi: 10.1186/s12879-021-06173-w (PMC8138111; doi:10.1186/s12879-021-06173-w)
Supplement: Supplementary file 5 — Additional file 5: Table S2. Emm75 qPCR and NanoDrop DNA readings. Comparison between DNA-only and single column combined extraction methods (RNA eluted first). Refer to Fig. 2b DNA readings. [file 12879_2021_6173_MOESM5_ESM.pdf]

DNA only extraction							Single-column simultaneous extraction					
**M75 load** CFU/mL	**Replicate**	***emm*75 qPCR** Ct value	**DNA estimation** ng/µL	**260/280**	**260/230**		**M75 load** CFU/mL	**Replicate**	***emm*75 qPCR** Ct value	**DNA estimation** ng/µL	**260/280**	**260/230**
10^7	A	17.59	28.5	1.70	0.07		10^7	A	20.09	20.4	1.74	0.73
	B	17.78	25.4	2.00	0.12			B	20.00	18.4	1.95	0.38
	C	17.66	20.6	2.03	0.38			C	20.37	20.2	1.88	0.58
10^5	A	24.48	5.7	1.90	0.10		10^5	A	30.55	7.0	1.59	0.30
	B	24.59	8.2	1.62	0.21			B	29.11	7.2	1.56	0.17
	C	24.90	7.2	1.65	0.28			C	26.59	5.5	1.73	0.10
10^3	A	31.42	7.6	2.02	0.04		10^3	A	32.61	4.6	1.63	0.37
	B	31.54	11.2	1.76	0.05			B	32.89	5.2	1.65	0.05
	C	31.38	8.7	1.51	0.05			C	32.69	6.6	1.55	0.50
10^2	A	32.94	9.1	1.67	0.05		10^2	A	No Cq	7.5	1.88	0.03
	B	34.15	9.9	1.60	0.05			B	No Cq	6.6	1.63	0.37
	C	33.92	8.8	1.44	0.04			C	No Cq	7.0	1.78	0.08
