# Supplementary material for: An emm-type specific qPCR to track bacterial load during experimental human Streptococcus pyogenes pharyngitis
Source: BMC Infect Dis. 2021 May 21;21:463. doi: 10.1186/s12879-021-06173-w (PMC8138111; doi:10.1186/s12879-021-06173-w)
Supplement: Supplementary file 6 — Additional file 6: Table S3. gyrA RT-qPCR and NanoDrop RNA readings. Comparison between RNA-only and single column combined extraction methods (RNA eluted first). Refer to Fig. 2b RNA readings. [file 12879_2021_6173_MOESM6_ESM.pdf]

RNA only extraction							Single-column simultaneous extraction					
**M75 load** CFU/mL	**Replicate**	**cDNA *gyrA* qPCR** Ct value	**RNA estimation** ng/µL	**260/280**	**260/230**		**M75 load** CFU/mL	**Replicate**	**cDNA *gyrA* qPCR** Ct value	**RNA estimation** ng/µL	**260/280**	**260/230**
10^7	A	25.61	3.9	1.13	0.18		10^7	A	22.48	3.2	0.91	0.16
	B	25.61	5.3	1.17	0.17			B	22.79	5.2	1.02	0.17
	C	25.01	4.9	1.16	0.13			C	24.30	5.5	0.96	0.19
10^5	A	35.07	4.3	1.05	0.11		10^5	A	36.60	2.3	0.74	0.09
	B	36.71	3.7	1.07	0.02			B	34.56	3.5	1.01	0.13
	C	34.23	2.5	0.88	0.08			C	34.86	3.5	0.88	0.13
10^3	A	No Cq	4.4	0.99	0.13		10^3	A	No Cq	2.2	0.71	0.12
	B	37.18	4.3	0.97	0.02			B	No Cq	3.0	0.93	0.02
	C	38.43	3.0	0.89	0.06			C	No Cq	4.7	1.08	0.19
10^2	A	No Cq	2.5	0.76	0.08		10^2	A	No Cq	1.0	0.49	0.03
	B	No Cq	2.8	0.78	0.10			B	No Cq	5.4	1.12	0.20
	C	35.24	2.6	0.70	0.11			C	No Cq	1.7	0.74	0.05
