# Supplementary material for: An emm-type specific qPCR to track bacterial load during experimental human Streptococcus pyogenes pharyngitis
Source: BMC Infect Dis. 2021 May 21;21:463. doi: 10.1186/s12879-021-06173-w (PMC8138111; doi:10.1186/s12879-021-06173-w)
Supplement: Supplementary file 7 — Additional file 7: Table S4. Detailed timeline of discrepancies between culture and qPCR results. Twenty swabs were positive by emm75 qPCR and negative by culture. These swabs were either collected within 48h after inoculation (N=4) or within 36h after initiation of antibiotic treatment (N=16). 0, pre challenge; +24, 24h post challenge; Abx, antibiotics; +1 w, 1week post discharge; +1m, 1month post discharge; +3m, 3months post discharge. [file 12879_2021_6173_MOESM7_ESM.pdf]

*emm*75 qPCR +	0	19	17	16	6	5	3	2	19	17	0	0	0
Culture +	0	17	16	15	6	5	3	2	12	8	0	0	0
qPCR +/culture -		2	1	1					7	9			
Timepoint	*0*	*+24*	*+36*	*+48*	*+60*	*+72*	*+84*	*+96*	*12h post Abx*	*Discharge*	*+1 w*	*+1 m*	*+3 m*
